# Pressure Sensitive Adhesive Tape: A Versatile Material Platform for Optical Sensors

**DOI:** 10.3390/s20185303

**Published:** 2020-09-16

**Authors:** Carlos Angulo Barrios

**Affiliations:** 1 Institute for Optoelectronic Systems and Microtechnology (ISOM), ETSI Telecomunicación, Universidad Politécnica de Madrid, Ciudad Universitaria s/n, 28040 Madrid, Spain; carlos.angulo.barrios@upm.es; 2 Department of Photonics and Bioengineering (TFB), ETSI Telecomunicación, Universidad Politécnica de Madrid, Ciudad Universitaria s/n, 28040 Madrid, Spain

**Keywords:** optical sensor, pressure sensitive adhesive tape, flexible photonics, polymer, waveguide, refractive index sensor, optomechanical sensor, vapor sensor

## Abstract

Pressure sensitive adhesive (PSA) tapes are a versatile, safe and easy-to-use solution for fastening, sealing, masking, or joining. They are widely employed in daily life, from domestic use to industrial applications in sectors such as construction and the automotive industry. In recent years, PSA tapes have found a place in the field of micro- and nanotechnology, particularly in contact transfer techniques where they can be used as either sacrificial layers or flexible substrates. As a consequence, various optical sensing configurations based on PSA tapes have been developed. In this paper, recent achievements related to the use of PSA tapes as functional and integral parts of optical sensors are reviewed. These include refractive index sensors, optomechanical sensors and vapor sensors.

## 1. Introduction

Pressure sensitive adhesive (PSA) tape (also known as self-adhesive, self-stick adhesive or sticky tape) can be defined as a continuous, relatively thin, flexible material with single or double sided coating that can adhere to a variety of substrates by applying gentle pressure without the need for solvent, heat, UV radiation or water for activation [1,2,3,4,5]. PSA tapes are widely used for sealing, bonding, attaching, communicating, identifying, insulating and protecting, and novel uses continue to be discovered. They have numerous advantages, for example, compared to traditional fastening systems, PSA tapes provide an easy to use and low cost solution that allows the use of thinner and lighter materials, the bonding of dissimilar materials without incompatibility concerns, vibration dampening and noise reduction, crack and corrosion prevention, removal of visible mechanical fasteners for cosmetic superiority, uniform thickness and gap filling, and reduced assembly time. Since adhesion is immediate, PSA tapes save time compared to liquid adhesives that require specific setups and/or long curing times. Several key markets have benefited from PSA tape usage including building and construction, the automotive, aerospace, marine, medical, appliances, electronics, and packaging industries, and they have been applied to the temporary fixation of components, solar modules, foam and gaskets, mounting emblems, and cable harnessing.

**Figure 1:**
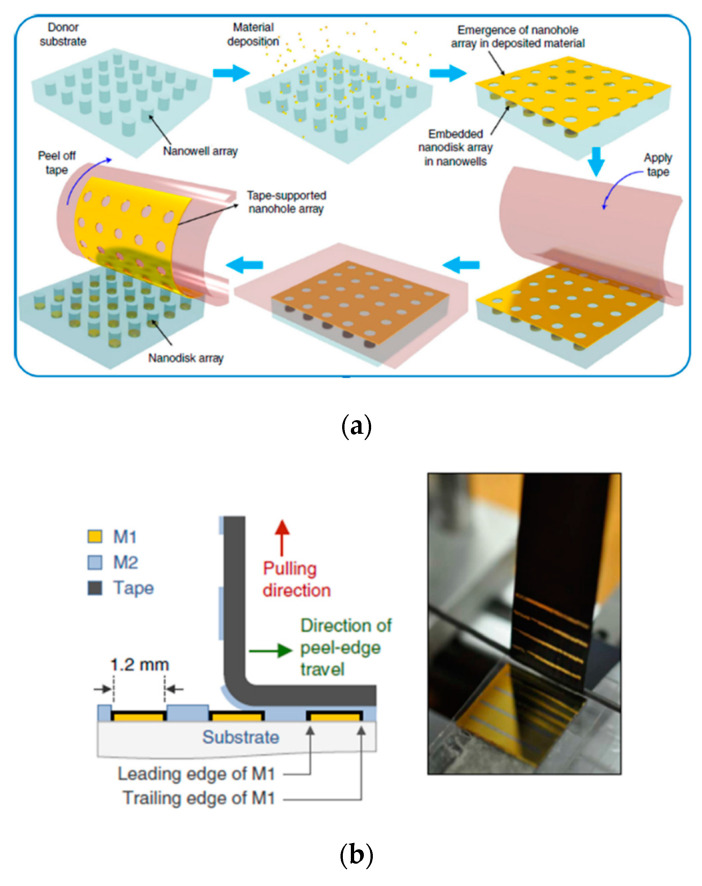
Adhesion or tape lithography. (**a**) Transfer of a nanopatterned film (Au nanohole array) to the surface of an adhesive tape. Reproduced from [22] under a Creative Commons Attribution 4.0 International License (http://creativecommons.org/licenses/by/4.0/). (**b**) Left: Schematics of selective nanostructured metal stripping by a pressure sensitive adhesive (PSA) tape. M1 and M2 stand for metal 1 and metal 2, respectively. Right: Photograph showing the tape after peeling; removed gold (M2) is visible on the adhesive side of the tape. Reproduced from [20] under a Creative Commons Attribution 3.0 Unported License (http://creativecommons.org/licenses/by/3.0/).

In recent years, the utilization of PSA tapes in the field of micro- and nanotechnology has gained much attention. In particular, PSA tapes have found great applicability in contact transfer techniques like transfer printing [6,7,8,9], where the flexibility and tackiness of the adhesive tapes permit the effective detachment of nanostructures from a donor substrate by a simple “stick and peel” procedure, and their incorporation into the sticky side of the tape. After peeling, the PSA tape can be used either as an intermediate transfer medium when the nanostructures are to be transferred to a different substrate, or as an integral part of the final device, for example, as a substrate for flexible devices. Unlike other polymeric materials that have been traditionally employed in flexible devices such as PDMS [10,11,12], the use of PSA tape does not require previous activation of the contacting surfaces. Relevant examples of nanotransfer printing using PSA tapes include the exfoliation of two-dimensional materials such as graphene [13] and MoS_2_ [14] and the transfer of carbon nanotubes [15] and nanowire devices [16,17]. PSA tapes have also been employed for planarizing nanopatterned substrates in order to create large-area nanogaps [18,19], and they are the key element in an innovative type of nanolithographic method: adhesion or tape nanolithography [20,21,22]. Figure 1 illustrates the operation of adhesion lithography based on PSA tape.

**Figure 2:**
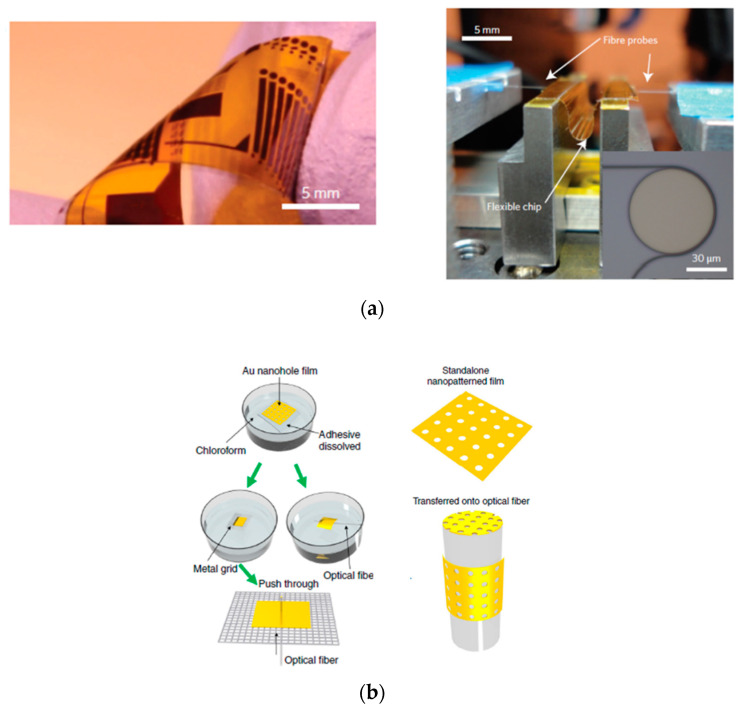
Examples of application of PSA tape to the construction of optical devices. (**a**) Left: Photograph of a flexible photonic chip built on a Kapton PSA tape substrate. Right: Photograph of a fiber end-fire testing set-up used for in situ measurement of optical transmission characteristics during mechanical bending of the flexible devices. Reprinted from [23] by permission from Springer Nature. (**b**) Schematic of a release-and-transfer process of an Au nanohole film to an optical fiber surface. Reproduced from [22] under a Creative Commons Attribution 4.0 International License (http://creativecommons.org/licenses/by/4.0/).

The development of optical and photonic devices and microsystems has been greatly enhanced by the application of PSA tape to micro- and nanotechnology. For instance, flexible photonic devices have been implemented using PSA tape as an integral functional component, either as a substrate [21,22,23,24] or as a waveguide [25,26,27,28]. Tape-supported nanopatterned films have also been successfully transferred to the tip and side surfaces of optical fibers by dissolving or detaching the supporting tape [22]. Figure 2 shows illustrative images of these applications. In addition, laser-microstructured double-sided biocompatible adhesive tapes have been employed as intermediated bonding layers for the development of microfluidic networks in opto-fluidic microsystems [29,30].

The implementation of optical sensors based on PSA tapes is a recent trend, and there are still few studies on these types of devices. However, the importance of the related reported works and the development potential of this technological approach is significant, which motivated this review paper on state-of-the-art optical devices based on PSA tapes for sensing applications. Section 2 briefly describes the main configurations and constituent materials of PSA tapes, with an emphasis on their optical, mechanical and thermal characteristics. In Section 3, particular applications of optical sensors based on PSA tapes are reviewed. These comprise refractive index sensors for liquids, optomechanical displacement sensors, and optical vapor sensors. Finally, some concluding remarks are given in Section 4.

## 2. PSA Tape Constructions and Materials

**Figure 3:**
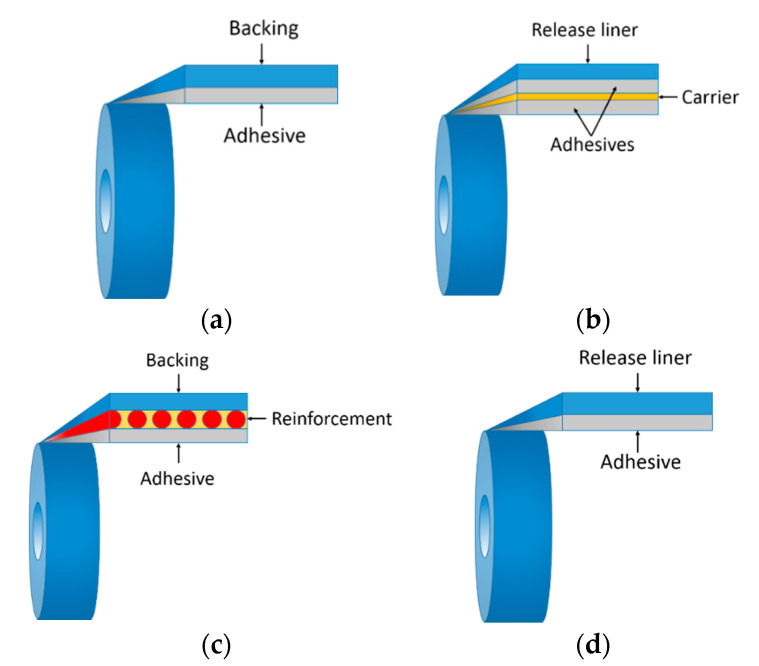
Schematics of typical PSA tape constructions [1]. (**a**) Single coated, (**b**) double coated, (**c**) reinforced, and (**d**) unsupported tape.

A PSA tape is basically composed of a backing (carrier) film and an adhesive layer. It is also common to find a release agent to the backing or a release liner on the adhesive in order to prevent the latter from sticking to the backing when the PSA tape is coiled in a roll. PSA tapes are typically available in four different product architectures (see Figure 3): single coated (the adhesive is applied to only one side of the backing), double coated (the adhesive is applied to both sides of the backing), reinforced (a reinforcement layer of woven or knitted cloth or glass strands is included) and unsupported (the adhesive is wound around a release liner). The usual thickness of both the backing and adhesive layers ranges from 20 µm to 200 µm.

The main adhesive types used in PSA tapes are rubber/resin, acrylic and silicone, with or without additives [1,2,31,32,33]. Rubber-based adhesives are formulated from mixtures of natural or synthetic rubber and resin. They provide high tack (ability to form a bond in a short time) and peel (force required to pull off the adhesive from the applied surface), and adhere well to several non-polar, low-energy surfaces. When compared to acrylics, rubber-based adhesives are typically less expensive; however, they are more affected when exposed to certain chemicals, UV radiation, or high temperatures. In addition, they are more susceptible to oxidation, which can make the adhesive darken, lose their tack, and become brittle.

Acrylic-based adhesives are formulated from cross-linked acrylic polymers (acrylates). They have high peel, tack and shear (resistance to shear stress), and exhibit excellent resistance to chemical, temperature and UV radiation exposure and oxidation. Acrylic adhesives are long-lasting and show good transparency and color stability, which are important characteristics from an optical sensor implementation point of view. They bond well to polar surfaces such as metal, glass, polyesters and polycarbonates. The disadvantages of acrylics as compared to rubber-based adhesives include poor adhesion to low-energy surfaces, such as polypropylene and polyethylene, and lower overall adhesion [1,2,31,32,33].

Silicone adhesives are less common and more expensive than rubber- and acrylic-based adhesives. They are formulated from silicone polymers and are usually destined for providing adhesion to silicon and other hard-to-adhere-to materials [1,2,31,32,33].

In principle, any material that is reasonably flat, thin and flexible can be used as a PSA tape backing. Typical backing materials are cloth, paper, metal, plastic (such as bi-axially oriented polypropylene (BOPP), polyvinyl chloride (PVC), and polyimide) or foam. The backing provides robustness and protects the adhesive from degradation due to environmental conditions such as humidity, temperature, and UV radiation. The majority of PSA tape suppliers offer a large variety of backing options so that the properties of the backing material can be selected according to the target use. In particular, plastic (polymeric) backings offer excellent optical, mechanical and thermal parameters for optical sensing applications, and are by far the most popular for implementing optical sensors based on PSA tapes.

### 2.1. Optical Properties of PSA Tape

Knowledge of the optical properties of the PSA tape materials is essential when adhesive tapes are intended to provide optical functionalities in devices and systems. For example, optical transparency and refractive index (n) are particularly important parameters for applications based on light transmission and waveguiding [34]. Table 1 and Table 2 show indicative values for relevant optical parameters of typical polymeric materials used for the construction of PSA tapes. The overall optical properties of adhesive tapes depend on the intrinsic constituent material properties and also on the manufacturing process and the structure of the tape. Three illustrative examples related to optical applications of PSA tapes found in both the literature and the technical data from tape manufacturers are described below:

(1) General-purpose #550 Scotch^™^ transparent tape, used in the fabrication of many optical sensors that will be reviewed in the next section, is a single-coated PSA 19-mm-wide tape consisting of a 30-µm-thick BOPP backing and a 20-µm-thick acrylic adhesive layer [35]. This sticky tape exhibits an internal transmittance, measured with a spectrophotometer in the spectral range 500–725 nm, which is nearly constant and equal to approximately 99.4%, and its refractive index at room temperature as measured by reflectance interferometry was found to be 1.45 [25].
(2) Luu et al. measured the optical properties of three types of general-purpose PSA tape employed in the implementation of microfluidic systems for optical interrogation [30]: Scotch^®^ permanent double sided tape (19.0 mm wide and 88.9 μm thick), Scotch^®^ gloss finish multitask tape (19.0 mm wide and 50.8 μm thick), and Scotch^®^ shipping packaging tape (47.7 mm wide and 78.7 μm thick). Scotch^®^ permanent double-sided tape is a cellulose film with an acrylate adhesive on both sides. Measurements of the refractive index were conducted with an Abbe refractometer. An experimental value of n = 1.51 at 630-nm-wavelength was found for all tapes. This result is in agreement with published data regarding the refractive index of cellulose (n = 1.47 at 630 nm), the baking material, averaged with that of styrene (n = 1.52 at 630 nm), the adhesive material. The three tapes had an absorption coefficient close to zero and low scattering coefficients (3–4 cm^−1^).
(3) 3M™ (Saint Paul, MN, USA) Optically Clear Laminating Adhesive 8146-x [36] is a family of unsupported PSA tapes consisting of an acrylic adhesive layer (thickness ranges from 25 µm to 125 µm, depending on the specific tape) sandwiched between two 70-µm-thick release liners made of polyester. The refractive index of these tapes is 1.474 at sodium D-line (589.3 nm) at 25 °C, measured with an Abbe refractometer, and exhibit light transmission >99% when corrected for reflection losses. These adhesive tapes are specifically designed to provide crystal clear, high reliability optical coupling and mechanical joining for various transparent materials. Typical applications include touch screens (for bonding film and glass laminates), transparent graphic overlays and optical management films for LCD.

A well-known optical property of many PSA tapes is birefringence [37,38,39,40,41,42]. This characteristic is provided by polymeric baking films (the adhesive is isotropic) and results predominantly from the manufacturing process. Stretching and molding processes involved in the production of the baking induce stress in the polymer, which appears as birefringence in the finished materials. A typical example is ordinary cellophane, which is birefringent and used as a common baking material in PSA tape (cellotape). The birefringence of cellophane tapes has been used in interference multilayer birefringent filters [38] and compensated polarized light microscopy [39].

### 2.2. Mechanical Properties of PSA Tape

Mechanical characteristics of adhesive tapes can also play a role in the design and fabrication of PSA tape-based optical sensors, particularly to those subjected to non-negligible mechanical stress and deformation. Common mechanical specifications for commercial PSA tapes are (peel) adhesion (typically to steel or glass surfaces), tensile strength and elongation at break. Table 1 and Table 2 collects some indicative values for the relevant mechanical parameters of PSA tape materials.

The adhesive material exhibits viscoelastic properties and has a low modulus, which makes it an effective strain-relieving agent in delamination processes [23]. The mechanical behavior of the adhesive material can be reduced to three fundamental and interconnected physical properties: tack, shear resistance and peel strength, which are strongly dependent on the bulk viscoelastic properties of the adhesive material system [55]. Less cross-linking results in higher tack, but more cross-linking provides higher shear. Peel adhesion and shear are a function of contact time, application pressure, and temperature. In general, as shear strength increases, tack and peel typically decrease. It is therefore difficult to maximize all three properties simultaneously. Thus, adhesives are formulated to meet peel, tack and shear requirements for particular applications.

By definition, PSA tapes are flexible, and therefore well-suited for implementing free-standing flexible optical structures. Typical polymeric backing materials used in general purpose PSA tapes, such as BOPP, PVC and polyimide, have good flexibility but limited stretchability. To fabricate stretchable photonic devices, specialized PSA tapes with elastomeric baking properties can be selected. For example, Scotch^®^ Stretchable Tape 8884 [56] is a 36-mm-wide and 130-µm-thick transparent stretchable tape, consisting of a low density polyethylene backing and synthetic rubber resin adhesive, that exhibits an adhesion to steel of 7.7 N/cm, a tensile strength equal to 35 N/cm and an elongation at break as high as 710%.

### 2.3. Temperature Effect

The optical and mechanical characteristics of both the backing and adhesive can be affected by temperature. At too-low temperatures, polymeric backings can become stiffer, and the viscoelastic adhesive can enter its glass state, becoming brittle and reducing adhesion [57]. At too-high temperatures, the adhesive material becomes more fluid and starts to flow as opposed to adhere. Both temperature extremes ultimately result in degradation of the mechanical performance the PSA tape. Additionally, these structural changes due to temperature can affect the material optical properties, such as transparency and the refractive index. Technical specifications usually provide the operating temperature range for PSA tapes, which is largely dependent on the type of adhesive. Within the operating temperature range, the combination of viscous and elastic properties of the PSA is well balanced, and the temperature-dependent behavior of the tape’s optical properties is mainly determined by the thermo-optic coefficient and the coefficient of thermal expansion of the tape’s polymeric materials, which are typically on the order of −10^−4^ K^−1^ and 10^−5^ K^−1^, respectively. A common operating temperature range for PSA tape application is between 15 °C and 35 °C [3]. Wider temperature ranges can be obtained by using Kapton^®^ tapes [49,51], which are made of Kapton^®^ polyimide backing film and silicone adhesive. Kapton tapes exhibit high thermal stability and are compatible with a wide temperature range from as low as −269 °C and as high as 260 °C. 

## 3. Examples of PSA Tape-Based Optical Sensors

### 3.1. Liquid Refractive Index Sensing

Measurement of the refractive index of liquids is of great importance for many applications, such as chemical and biochemical analysis, environmental monitoring and the food processing industry. Both guided-wave and free-space optical schemes can be used to implement refractive index optical sensors, and PSA tapes have been employed in both cases.

#### 3.1.1. Guided-Wave Refractive Index Optical Sensors

Optical fibers based on glass or polymers have been used extensively for refractive index sensing. These sensors are compact and can be designed for distributed [58,59] or tip-based [60,61] sensing. In order to increase the interaction of the optical field with the liquid analyte, that is, the refractive index sensitivity, optical fiber sensors can adopt different configurations, such as stripped [61] tapered [62], D-shaped [63,64,65,66], microstructured [67,68], and U-shaped [69,70] fibers. As compared to glass fibers, plastic optical fibers (POFs) are easy to handle and cost-effective. In addition, their multimode characteristics make POFs more suitable for intensity modulation schemes [71,72].

**Figure 4:**
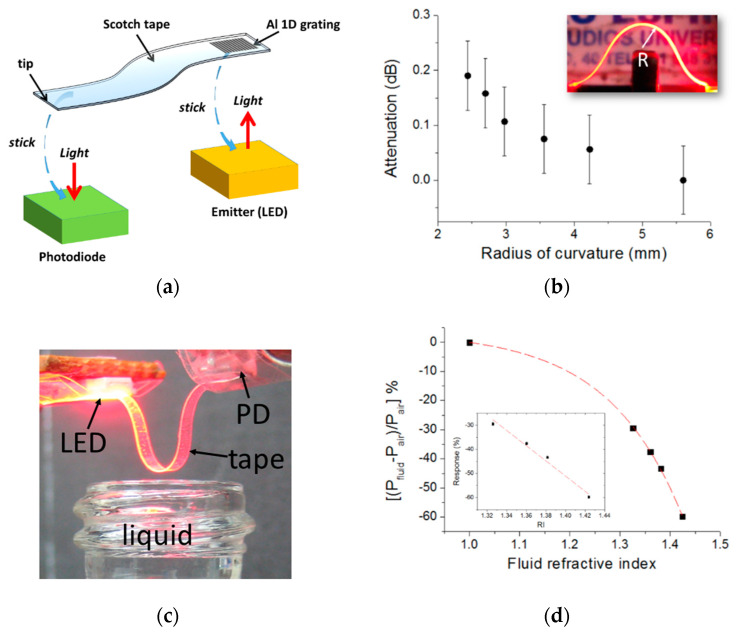
(**a**) Schematics of the assembly of a liquid refractive index optical sensor based on a Scotch tape waveguide with an integrated aluminum one-dimensional (1D) grating coupler. (**b**) Bending attenuation of a 24.7-mm-long tape waveguide at different radii of curvature (R). (**c**) U-shaped tape waveguide ready to be immersed in a liquid sample to measure its refractive index. (**d**) Calibration curve of the tape refractive index sensor. Inset shows the measured values in the refractive index range 1.326–1.424. Reprinted from [27].

Inspired by the aforementioned advantages of POFs, Barrios [27] proposed and demonstrated a plastic free-standing waveguide made of a Scotch tape for liquid refractive index sensing. The flexible waveguide contained an integrated aluminum grating coupler [25,26], which, when stuck on the radiative surface of a light emitting device, allowed light to be coupled in and transmitted through the tape waveguide, whose tip end was, in turn, directly adhered onto the photosensitive surface of a photodetector (Figure 4a). The (de)coupling approach exhibited high alignment tolerances that permitted the formation of a rapid, flexible optical connection between surface-normal optoelectronic devices without the need of specialized equipment. A 24.7-mm-long 180° bent Scotch tape waveguide was tested as an intrinsic, intensity-based refractive index sensor based on the dependence of the waveguide bending losses with the refractive index of the surrounding medium. The U-shaped waveguide was immersed in different liquids, and the sensor response showed exponential behavior with the liquid refractive index (Figure 4d). In particular, the sensitivity for refractive index values around 1.424 (cyclohexane) was −419 (%)/RIU, where RIU stands for refractive index unit. This meant a refractive index resolution (limit of detection) of 7 × 10^−4^ RIU. This performance compares well to other intrinsic, intensity-based optical fiber sensors [73,74].

#### 3.1.2. Free-Space Interrogated Refractive Index Optical Sensors

Optical sensing schemes based on free-space optical interrogation avoid some of the drawbacks related to guided-wave configurations, such as optical coupling complexity. There are multiple free-space configurations for liquid refractive index sensing that are mainly based on nanostructured surfaces such as plasmonic nanohole arrays (NHAs) [75,76,77,78]. A metal NHA can exhibit optical spectral features in both reflection and transmission related to surface plasmon effects that are sensitive to the refractive index of the medium in which the metallic nanostructure is immersed. In particular, surface plasmon polariton (SSP) block waves in a metal grating occur at wavelengths, *λ_SPP_*, given by:

(1) $$\lambda_{SPP}\approx \frac{a}{\sqrt{i^{2}+j^{2}}}\sqrt{\frac{n_{d}^{2}\varepsilon_{m}}{n_{d}^{2}+\varepsilon_{m}},}$$

where a is the array period, *i* and *j* are the grating orders, *ε_m_* is the dielectric function of the metal and *n_d_* is the refractive index of the surrounding dielectric medium. Refractometric NHA sensors have been primarily fabricated on rigid substrates such as glass; however, there are remarkable demonstrations on flexible supports such as PDMS [22], polycarbonate [78], and, recently, PSA tapes.

**Figure 5:**
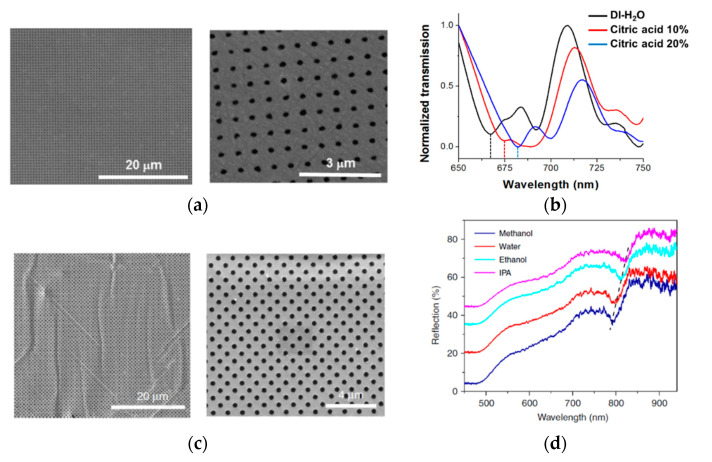
Scanning electron microscope photographs of a 625-nm-period Al nanohole array (NHA) (**a**) and a 600-nm-period Au NHA (**c**) transferred onto Scotch adhesive tapes. Both nanostructured surfaces exhibit excellent uniformity over large areas. Spectral transmission (**b**) and reflection (**d**) of the NHAs shown in (**a**,**c**), respectively, when immersed in liquid solutions having different refractive indices. Spectral features used as the refractive index sensor response are indicated with dotted lines in both (**b**,**d**). (**a**,**b**) are reproduced from [21] with permission from the Royal Society of Chemistry (https://www.rsc.org/journals-books-databases/journal-authors-reviewers/licences-copyright-permissions/). (**c**,**d**) are reproduced from [22] under a Creative Commons Attribution 4.0 International License (http://creativecommons.org/licenses/by/4.0/).

The use of a Scotch tape as a substrate for a metal NHA operating as a refractometric sensor was pioneered by Barrios et al. [21]. These authors fabricated a square-lattice 625-nm-period aluminum NHA on a general-purpose Scotch tape (Figure 5a) by direct transfer from a polycarbonate CD donor substrate. The device was tested as a refractive index sensor by immersing it into different aqueous solutions of citric acid. In the refractive index range from 1 to 1.36, a linear response was measured with a sensitivity of 477 nm/RIU (Figure 5b). A subsequent demonstration of a tape-based metal NHA refractometric sensor was achieved by Wang et al. [22]. These authors implemented a square-lattice 600-nm-period gold NHA on a Scotch tape (Figure 5c) by transfer from a PDMS donor substrate. Through immersion in liquids with different refractive indices (Figure 5d), the device exhibited a linear response with a sensitivity of 590 nm/RIU in the refractive index range from 1 to 1.38. Although both PSA tape-based Al and Au NHAs refractometric sensors presented some nanoscale cracks that were generated during the tape transfer process, which can decrease the Q factor of the device resonances, the experimental sensitivities are comparable to similar devices fabricated on rigid substrates. Therefore, tape supported metal NHAs offer great potential for biosensing that relies on refractive index changes in the vicinity of the nanoholes due to specific analyte-ligand binding events.

### 3.2. Opto-Mechanical Sensing

Opto-mechanical sensors can be used for a wide variety of applications, including the detection and/or monitoring of pressure [79], force [80,81], mass [82] biomolecules [83], temperature [84], magnetic field [85], displacement [86], strain [87] and acceleration [88]. Many of these optical sensors are based on a cantilever [89], whose bending or vibration frequency state changes in response to a physical or (bio)chemical magnitude variation. The cantilever state can be monitored optically by different means such as optical beam deflection [90], interferometry [91], interdigitated transducer deflection [92], waveguide coupling [93], integrated Bragg grating [94] and diffraction grating coupling [95]. Most cantilever-based sensors rely on cantilevers with micrometric or nanometric dimensions (micro/nanocantilevers); however, opto-mechanical sensing configurations based on several-millimeters long cantilevers have also found great applicability. These are mainly based on glass or plastic optical fibers that act as cantilevers [87,88].

**Figure 6:**
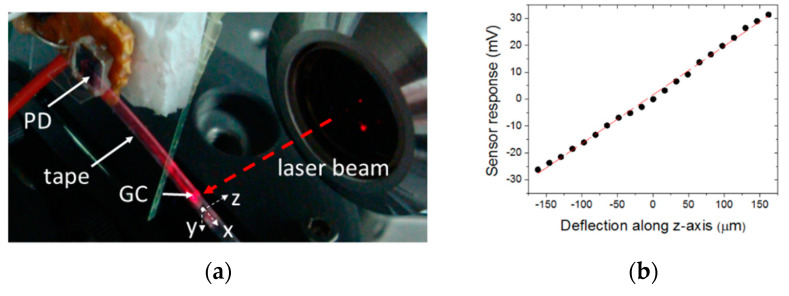
(**a**) Photograph of the fabricated tape waveguide cantilever with an integrated grating coupler (GC) [28]. The GC-free waveguide tip end is anchored onto a photodiode (PD), and the tip containing the embedded GC deflects along the z-axis. (**b**) Experimental cantilever response as a function of the tip-grating deflection [28] and a linear fit of the measured data (red line).

In the latter context, the guiding-wave structure described in Section 3.1.1 was employed to demonstrate a deflection sensor [28] using the Scotch tape waveguide as a cantilever with an integrated metal grating coupler at its deflecting end. In such a configuration (Figure 6a), light impinging the embedded grating was coupled into the tape waveguide and guided to a fixed photodetector, which acted as both the cantilever support and optical power-photocurrent converter. The amount of power measured by the photodetector is dependent on the overlap of the incident light beam spot with the grating coupler; this overlap, in turn, is a function of the cantilever deflection. Experimental characterization of a 14.85-mm-long cantilever sensor showed a linear working range of two decades (Figure 6b) and a deflection resolution of 1.7 µm, which was limited by the noise of the light source (semiconductor laser). The reported noise analysis indicated that sub-nanometric resolution is feasible by using, for example, differential compensation techniques [96] and laser beam stabilizers [97].

**Figure 7:**
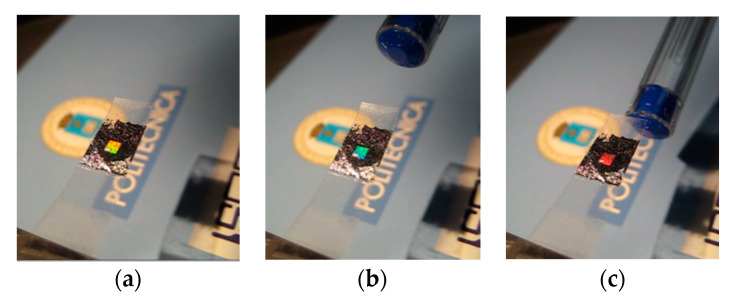
An electrostatic-opto-mechanical sensor [21]. (**a**) A Scotch tape cantilever with an integrated Al diffraction grating. The tape is electrically charged by peeling it off. When a plastic pen is placed close to the charged tape (**b**,**c**), opposite charges are induced in the pen. Electrostatic forces between the tape and the pen result in bending of the cantilever, which is observed as an iridescent color change of the Al diffraction grating.

Another transduction mechanism that can be used in the described Scotch tape deflection sensor is based on the color exhibited by the integrated diffraction grating when it is illuminated by white light. This color will depend on the angle of incidence of the illuminating light beam, which is a function of the cantilever tape deflection. This principle is illustrated in Figure 7, which shows an Al diffraction grating integrated into a Scotch tape cantilever operating as an electrostatic-opto-mechanical sensor [21]. This device uses the triboelectric effect of the adhesive tape, which can be electrically charged by peeling it off, to optically detect the presence of permanent or induced electrical charges in its vicinity. The readout equipment can combine a broadband light source, a high-resolution imaging spectrometer and a low-noise CCD camera to extract spectral information [98].

Fiber Bragg grating (FBG) strain sensors are an important type of optomechanical sensor [99,100]. In these devices the effective refractive index of the fiber core and the spatial periodicity of the grating are both affected by changes in strain due to the strain-optic effect. This produces a shift in the Bragg wavelength, which is related to variations in the fiber strain. A relevant application of these optical strain sensors is structural health monitoring based on the collection of ultrasonic waves [101,102,103,104]. For this purpose, adhesives are typically used for bonding the strain measuring fiber and the host structure in order to assure permanent mechanical coupling between both elements, thus avoiding losses due to sliding. The adhesive not only provides fixing but also plays a key role in the sensor performance since longitudinal and transverse displacements at the surface produced by Lamb waves in the structure are transferred to the optical fiber through the adhesive, leading to axial strain along the FBG optical fiber axis [102,103]. In particular, the symmetric S0 or antisymmetric A0 Lamb waves are coupled to guided L01 waves in an optical fiber at the adhesive bond location, resulting in L01 modes of equal amplitudes propagating in both directions along the optical fiber. Wee et al. [104] demonstrated that when the adhesive bond is replaced with adhesive tape the S0 Lamb waves couple to the L01 modes with a preferential direction. The directional coupling that is produced by the adhesive tape could be applied to design multiplexed FBG sensor arrays with specified signal pathways through the optical fiber networks.

### 3.3. Volatile Organic Compound Vapor Sensing

The detection of volatile organic compounds (VOCs) is a subject of great interest with applications in a variety of fields ranging from environmental monitoring to chemical and food industries. VOCs can be flammable and explosive above a certain concentration; therefore, optical sensors are particularly well suited for VOC detection as electrical spark hazards can be largely eliminated. An optical vapor sensor typically consists of a material, such as a polymer, that is sensitive to the vapor analyte integrated with an optical transducer, such as a Fabry-Perot cavity. Many examples can be found in the literature of optical vapor sensors that are a result of combining many different sensing materials with optical transducers, for example, [105,106,107,108,109].

**Figure 8:**
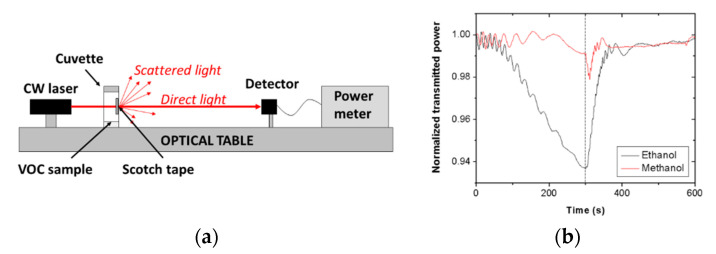
(**a**) Schematic diagram of the setup used for optical interrogation of a Scotch tape vapor sensor. (**b**) Normalized transmitted optical power at 635 nm wavelength through a piece of Scotch tape subjected to ethanol (black line) and methanol (red line) vapor exposure–exhaust measurements. Reproduced from [110].

Recently, a simple optical vapor sensor based on single-coated transparent Scotch tape for the detection of methanol and ethanol vapors was investigated [110]. The adhesive side of the tape was exposed to ethanol and methanol vapors, and the tape was simultaneously illuminated by a 635-nm-wavelength laser beam (Figure 8a). The transmitted power was dependent on the vapor type and exposition time, which was attributed to light scattering and polymer swelling variations occurring in the tape that are produced by the exposure of the latter to the different vapors. Thus, the Scotch tape played the roles of both the sensing material and the transducing element of the chemical sensor. The adhesive tape exhibited high selectivity for ethanol vapor over methanol vapor (Figure 8b), a linear detection range of 0–100 vol% for ethanol-methanol mixtures, and detection limits of 8.8 vol% ethanol and 17.6 vol% methanol. For comparison, detection limits reported for enzymatic biosensors [111] were 3 vol% liquid methanol and 23 vol% liquid ethanol, and 0.5–2.1 vol% liquid ethanol for fluorescence probes [112]. Hence, it was concluded that the studied Scotch tape could be used as a simple and cost-effective disposable sensor for easy integration in optical platforms targeting ethanol–methanol mixture testing, either on-the-spot or remotely. Note that, besides the reported free-space interrogation scheme, a guiding-wave version of the sensor is also feasible using the Scotch tape as a waveguide, similar to VOC optical fiber sensors [106].

## 4. Discussions

Commercial PSA tapes are ubiquitous in many research laboratories, where they are mainly employed as a cheap, quick, versatile and easy-to-use solution for fastening, sealing, masking and joining. The possibility of using ready-available PSA tapes for the implementation of optical sensors, either as a manufacturing tool or as a functional component of the device, can therefore greatly simplify the work of researchers, especially in the demonstration of prototypes or proof-of-concept devices.

From an industrial perspective, PSA tapes can be provided at low cost in large quantities and in different finished formats, typically in a roll form, which should facilitate its incorporation in assembly lines for mass production of optical sensors. Additionally, the constituent materials of conventional adhesive tapes are well known and so is the related technology to process them. Nevertheless, for commercial use of the reviewed optical sensors based on PSA tapes, sensor performance parameters such as repeatability and reproducibility need to be studied to a greater extent.

Future research could be directed towards further exploiting the opto-mechanical properties of both general-use and application-oriented PSA tapes, investigating the surface functionalization of adhesive tapes for the implementation of optical biosensors, and the exploration of new sensor configurations and applications based on, for example, the addition of nanomaterials (nanoparticles, nanowires and 2D materials) to the adhesive layer of the tapes, and the capability of PSA tapes to emit terahertz and optical radiation [113].

## Figures and Tables

**Table 1:** Indicative values of optical, mechanical and thermal parameters of typical polymeric backing materials used in transparent pressure sensitive adhesive tapes. BOPP = biaxially oriented polypropylene; PVC = polyvinyl chloride; CTE = coefficient of thermal expansion.

Material	Refractive Index	Birefrin-Gence	Tensile Strength at Break (N/cm)	Elongation at Break (%)	CTE (K^−1^)	Thermal Conductivity (W m^−1^ K^−1^)
BOPP	1.50 @ 589.3 nm [41]	~0.01 ^a^ [41,42]	40 [35]	30 [35] 160 [43]	1.0–1.8 × 10^−5^ [44]	0.1–0.22 [44]
PVC	1.54 @ 589.3 nm [45]	0.027 ^b^ [46]	47.5 [47]	60 [47]	5 × 10^−5^ [48]	0.14–0.17 [48]
Polyimide	1.70 @ 589.3 nm [49]	0.0263 ^b^ [50]	57.8 [51]	62 [51]	2 × 10^−5^ [49]	0.120 [49]
Cellophane	1.47 @ 632.8 nm [40]	0.0041 ^b^ [40] 0.0077 ^b^ [37]	40.2 [52]	15 [52]	8 × 10^−5^ [53]	0.0600 [53]

^a^ Calculated as (n_x_ + n_y_)/2 − n_z_, where n_x_ and n_y_ are the refractive indices measured in the plane of the film and n_z_ the refractive index through the thickness of the film. ^b^ Calculated as |n_e_ − n_o_|, where n_e_ and n_o_ are the refractive indices of the material for the extraordinary and ordinary waves, respectively.

**Table 2:** Indicative optical and mechanical parameters of adhesive materials used in pressure sensitive adhesive tapes. From [54]. Values can vary depending on the particular product.

Material	Refractive Index	Haze ^a^ (%)	Transmission (%)	Peel Adhesion ^b^ (N/cm)
Resin/Rubber	1.52	0.04	99.8	6.7 to Glass
Acrylate	1.47	<0.5	>99	5.8 to Steel
Silicone	1.41	1.0	99	6.7–7.8 to Glass

^a^ Amount of light that is subjected to Wide Angle Scattering (at an angle greater than 2.5° from normal). ^b^ For a 25 mm wide tape.
